# Top-down contingent feature-specific orienting with and without awareness of the visual input

**DOI:** 10.2478/v10053-008-0087-z

**Published:** 2011-12-22

**Authors:** Ulrich Ansorge, Gernot Horstmann, Ingrid Scharlau

**Affiliations:** 1 Faculty of Psychology, University of Vienna, Austria; 2 Institute of Cognitive Science, University of Osnabrück, Germany; 3 Department of Psychology, University of Bielefeld, Germany; 4 Faculty of Cultural Sciences, University of Paderborn, Germany

**Keywords:** vision, masking, attention, top-down contingent capture

## Abstract

In the present article, the role of endogenous feature-specific orienting for conscious and unconscious vision is reviewed. We start with an overview of orienting. We proceed with a review of masking research, and the definition of the criteria of experimental protocols that demonstrate endogenous and exogenous orienting, respectively. Against this background of criteria, we assess studies of unconscious orienting and come to the conclusion that so far studies of unconscious orienting demonstrated endogenous feature-specific orienting. The review closes with a discussion of the role of unconscious orienting in action control.

## Introduction

 Active vision – orienting towards visual stimulation – has evolved over hundreds of millions of years. Even organisms as simple as nematodes or protozoa show phototaxis, that is, they move towards light sources. How is orienting brought about? Is awareness of the visual input (i.e., *conscious vision*) necessary for it? We do not know whether simple organisms, such as Nematodes, have faint precursors of awareness. Given that their nervous systems are not very complex, they probably orient without awareness and conscious vision. However, to date, only introspective report taps into awareness, and we do not know how to assess awareness objectively so that we can tweak it in animals. Fortunately, the hypothesis of unconscious visual orienting can also be tested with humans. There are now numerous articles demonstrating orienting of attention without awareness (cf. McCormick, 1997; Scharlau, 2007). For example, McCormick (1997) presented his participants with the target of a reaction time task either to the left or to the right of the screen center. Participants did not know at which position the target was shown. Prior to the target, a brief cue appeared at one of the two possible target positions. As a consequence of the participants’ covertly orienting towards the cues, target responses were facilitated when cue and target appeared in the same position rather than in different positions. This was the case even though the participants remained un-aware of the cue due to a low cue-background contrast. 

Yet, what does it mean that humans can orient towards visual stimuli of which they remain unaware? Does it mean that *unconscious orienting*, as we will call this ability, is stimulus-driven or exogenous as McCormick concluded (see also Mulckhuyse & Theeuwes, 2010b)? Indeed, some unconscious mental processes can run off in a stimulus-driven fashion. For these exogenous processes to run off, the human agent does not need to exert will. Think of a ray of light impinging on the retina. Via its photonic energy, the ray will start a cascade of events in an exogenous way, from transduction of light into nervous energy, up to phenomenal visual awareness. Even the strength of the sensory representations is governed by precise laws. Up to stimulus durations of about 60 ms, luminance and duration of a visual stimulus are integrated in a linear fashion for lightness perception (Bloch, 1885). As humans, we do not have anything to do to initiate these visual *sensory processes* but to literally let them happen.

Yet, caution is advised: The fact that exogenous processes can be unconscious does not necessarily mean that unconscious processes must be exogenous. Empirically, this has been shown to be particularly true of unconscious visual orienting (cf. Ansorge & Neumann, 2005; Woodman & Luck, 2003), that is, the selection of one spatial position from the environment.

Orienting as defined by spatial selection can be testified in one of two ways. First, it can be reflected in behavioral preferences for one position over the other, for instance, when the eyes turn toward one particular position in space (Posner, 1980). Second, it can show up in perceptual performance changes, in the form of a boosted detection, discrimination, or identification of stimuli at one particular position in space (Posner, 1980). In the latter case, it is not necessary that the eyes move towards the position. It is sufficient to covertly shift attention (Helmholtz, 1895, 1896).

In our review below we will discuss two ways of unconscious orienting. One way is endogenous (Ansorge, Kiss, & Eimer, 2009). It starts with the selection of one visual feature and proceeds with the selection of the location of this feature. This way of orienting is endogenous or top-down controlled because initially participants intentionally set up the feature templates by which they search for task-relevant stimuli. Only visual stimuli that match these templates do then attract attention and lead to orienting. The other way is exogenous. It starts with a feature-unspecific selection of one visual location and proceeds with the selection of visual features from this location (McCormick, 1997; Mulckhuyse & Theeuwes, 2010b).

In the present review, we focus primarily on backward masking research. *Backward masking* is a powerful method to reduce stimulus visibility (cf. Breitmeyer, 1984). If two brief visual stimuli are successively presented at the same or adjacent positions but with a short interval of about 30-100 ms between them, the first of these stimuli – henceforth the “prime” – suffers from masking by the second stimulus – henceforth the “mask”: Some of the prime’s features (i.e., its shape and its color) can barely be seen (cf. Alpern, 1953; Breitmeyer, 1984; Breitmeyer & Ogmen, 2006; Reeves, 1981; Stigler, 1910).

The major advantage of the method of masking is that it can be used in healthy volunteers to study unconscious vision. Besides reviewing masking studies, we will also occasionally refer to studies using alternative methods to prevent conscious vision and secure unconscious vision in healthy participants (e.g., McCormick, 1997; Mulckhuyse, Talsma, & Theeuwes, 2007). With very few exceptions (e.g., Goodale & Milner, 1992; Weiskrantz, 1986), however, we will not systematically review studies with patients with neuropsychological impairments of conscious vision, because of the uncertainties associated with this kind of evidence, like substitution of impaired processing routes by alternative processing mechanisms in patients.

## What is the function of orienting?

A vast amount of research suggests that one major function of orienting is the facilitation of perception of visual information at the selected position (e.g., Posner, 1980; Titchener, 1908; Treisman & Gelade, 1980). In the typical experiment, one of several relevant visual target stimuli is presented at one of several spatial positions. Participants neither know at which position the next target will be shown, nor do they know the target’s exact identity. In this situation, prior orienting of the participants towards one of the potential target positions improves the participants’ accuracy of discriminating a target at this location and their speed of responding to it (e.g., Jonides, 1981; Müller & Rabbitt, 1989; Yantis, 1988, 1993; Yantis & Jonides, 1984). For instance, target discrimination is better when a small cue is presented shortly before target onset and at the same position (SP) as the target than when the cue indicates a different position (DP) than the target (e.g., Jonides, 1981).

## How is orienting carried out?

### Orienting before feature selection

#### Abrupt onset singletons

How is orienting carried out? One possibility is that visual orienting proceeds as, first, an exogenously-driven selection of a position and, second, a subsequent selection of visual features from this position. Three exogenous principles have been advocated for the initiation of orienting. One principle is exogenous or stimulus-driven orienting towards onset singletons (Breitmeyer & Ganz, 1976; Jonides, 1981). *An onset singleton* is a single stimulus that newly appears at a particular point in time while other stimulus or background elements are stationary at the very same moment. Some researchers believed that onset singletons were of special relevance during evolution because the quick detection of a suddenly appearing object in the visual field allowed orienting so that subsequently feature and identity information about the onset stimulus could be evaluated (cf. Breitmeyer & Ganz, 1976). In this manner, the visual system would readily orient to a changing visual input as a source of potential harm, for example, to avoid being struck by a falling rock.

Indeed, participants can very quickly orient towards abrupt onset cues. Cueing effects of abrupt onset singleton cues, with better performance in SP than DP conditions, require only very brief cue-target intervals of a few milliseconds (cf. Müller & Rabbitt, 1989; Nakayama & Mackeben, 1989). In addition, abrupt onset cueing is found for uninformative cues where SP and DP conditions occur unpredictably, for instance, and cue and target position are uncorrelated or only weakly correlated (cf. Jonides, 1981; Lambert, Spencer, & Mohindra, 1987). In fact, participants even have problems ignoring an uninformative abrupt onset cue, if asked to do so (cf. Jonides, 1981; Remington, Johnston, & Yantis, 1992). In contrast to this, if a symbolic cue is presented – that is, a cue such as an arrow in the center of the screen pointing towards one peripheral target position, so that a feature, such as the cue’s shape, must be used for orienting – participants need to have some confidence in the information value of the cue for predicting the target position, and participants need longer cue-target intervals to use the cue (e.g., Müller & Rabbitt, 1989). It therefore seems that abrupt onset singletons are special, exogenously capture attention and drive orienting, regardless of the goals of the participants.

#### Singleton or salience-driven capture

The second exogenous orienting principle that has been advocated generalizes this idea. Some authors assumed an orienting mechanism sensitive to singletons, or regions of high local feature contrast – that is, salient regions (cf. Bergen & Julesz, 1983; Itti & Koch, 2001; Theeuwes, 1992). This principle considers local visual feature contrasts in at least color, luminance, and orientation as crucial input for exogenous visual orienting. Exogenous orienting is instigated if the local feature contrast at one position is considerably stronger than feature contrasts at all alternative positions (e.g., Parkhurst, Law, & Niebur, 2002). According to this view, an onset singleton driving attention is just a special case of stimulus-driven orienting by strong local feature contrasts.

This assumption is supported, for example, by the examination of eye fixation patterns. Fixations are the phases in which the gaze rests at a particular image location. Under free viewing of 2D images of natural scenes, fixations on regions with high local feature contrast (i.e., singletons) are far more frequent than expected on the basis of a chance distribution of all fixations (cf. Parkhurst et al., 2002). Feature singletons or feature contrast maxima are also believed to help orienting towards interesting visual positions, such as object edges, for the subsequent selection of the particular identity of the visual features from these positions (cf. Mulckhuyse & Theeuwes, 2010b; Theeuwes, 2010).

#### Capture by overlearned symbols

Researchers argued for a third exogenous orienting principle by overlearned symbols, such as seen eye gaze direction (cf. Langton & Bruce, 1999), pointing directions of arrows (e.g. Eimer, 1997; Tipples, 2002), and even spatial words, like *left* or *right* (cf. Hommel, Pratt, Colzato, & Godijn, 2001). It is assumed that humans have so much experience with the use of the spatial meaning of these stimuli for attention shifts that these stimuli can trigger attention shifts without any prior intention on the side of the observer. In line with this assumption, if used as cues, all of these stimuli trigger attention shifts even if they are irrelevant and completely uncorrelated with the position of a relevant target stimulus (Eimer, 1997; Friesen & Kingston, 1998; Hommel et al., 2001).

#### Masking

The sequence of exogenous orienting preceding visual feature selection is also supported by masking research. Masking research suggests that conscious perception of visual features depends on prior orienting towards a visual stimulus. Prime features, such as prime shape and prime color, are lost for conscious perception if a mask substitutes the prime in the critical time window between prime onset and the conclusion of orienting towards the prime (cf. Bachmann, 1999; Enns, 2004; Neumann & Scharlau, 2007a, 2007b).

In line with the assumption that the crucial mediating variable here is the time to shift attention to the prime, masking of visual prime features, such as its color or shape, becomes weaker for primes (and masks) inside the focus of attention (cf. Bridgeman & Leff, 1979; Enns, 2004). Also, in line with the assumption that abrupt onsets are special in that they are available before additional features of the same stimulus can be selected, the prime’s temporal onset and its spatial position are usually spared from masking (cf. Bachmann, 1999; Fehrer & Raab, 1962; Schiller & Chorover, 1966).

This temporal onset sparing is reflected in perceptual latency priming (Bachmann, 1999; Scharlau, 2007; Scharlau & Neumann, 2003a, 2003b). When a primed mask and a similar unprimed stimulus are presented in perfect synchrony, humans often see a sequence of events: The primed mask is seen as temporally preceding the unprimed stimulus. This perception even arises if the primed mask actually trails the other stimulus by a short amount of time (Scharlau, 2007). This is due to prior entry (cf. Titchener, 1908): Orienting towards the prime saves the time of the otherwise necessary orienting towards the subsequent mask. The time that is saved to see the primed mask (i.e., the amount of perceptual latency priming) is proportional to the prime-mask interval up to the time which is needed for the completion of the attention shift towards the mask (above this, the effect slowly declines to small values; cf. Scharlau, Ansorge, & Horstmann, 2006). Hence, information about prime onset time survives masking.

Spatial onset sparing is also reflected in perceptual latency priming (Scharlau, 2004b). The comparison stimulus does not benefit from a prime at a different position, and perceptual latency priming can show up at two separate, primed locations, but stimuli at positions in between two primed locations, will not benefit from perceptual latency priming (Scharlau 2004a). This indicates that both the information about the prime position and its onset time are available despite the masking of the prime, and that prime onset and prime position jointly account for perceptual latency priming.

#### Conclusion

Cueing research, fixation behavior, and masking research suggest that humans select information about an abruptly onsetting visual stimulus’ position prior to the conscious perception of some other features of this stimulus. In addition, masking research indicated that orienting could be a crucial prerequisite of the conscious perception of additional features of a prime stimulus, such as its color or its shape. We will next review, however, that dissociations between feature visibility and feature selection suggest that features, such as prime color and prime shape, can also be selected prior to conscious perception and prior to orienting proper and that therefore the exogenous-orienting mechanisms of abrupt-onset capture and singleton capture have been called into question.

### Visual feature selection before orienting

#### Dissociations between feature visibility and feature selection

Numerous masked-priming studies have demonstrated that the visual features of a masked prime, such as its precise shape or its color, are selected before orienting has been completed. Even if participants do not complete orienting towards a prime before mask onset and the prime is hence not seen, prime shape and prime color can influence endogenously controlled behavior (e.g., Neumann & Klotz, 1994). The corresponding evidence takes the form of dissociations between feature selection and feature visibility. These dissociations shed light on the sequence of events during orienting. In light of these dissociations, it is a theoretical possibility (to be evaluated further below) that the sequence of events in orienting is reverse to the usually assumed sequence where orienting obligatorily leads feature selection, and that actually feature selection can precede orienting and that orienting can in fact even be conditional on feature selection, during both conscious and unconscious orienting.

 Many studies showed that masked prime features are selected prior to their conscious perception and, hence, prior to the completion of orienting towards these primes.^1^ For instance, in a masked-priming study, Neumann and Klotz (1994) demonstrated that different prime shapes were selected and triggered their associated responses, even if these prime shapes were so strongly masked as to be completely blocked from conscious perception. Neumann and Klotz presented as targets a square on the left and a diamond on the right, or a square on the right and a diamond on the left. Their participants had to respond to the position of the square. They had to press a right key if the square was on the right and a left key if the square was on the left. In the same conditions, the target and the distractor were also used as masks for primes that were presented just prior to the target and distractor. The prime pair also consisted of a square and a diamond but of smaller size, such that the primes were backward-masked by the surrounding mask contours. In congruent conditions, the square-shaped prime and target were presented at the same position. For instance, the prime square was presented on the right and so was the target square. In incongruent conditions, the prime square was presented on the opposite side of the target. For instance, the prime square was presented on the right but the target square was shown on the left. Under these conditions, responses were faster in congruent than incongruent conditions. This was observed although the prime could not be seen as demonstrated in separated prime detection tasks. 

This basic dissociation between zero prime shape visibility – as a consequence of a lack of orienting – and prime shape selection – as concluded from the congruence effect of the prime – means that feature selection (here shape selection) is evidently a predecessor of orienting. This dissociation has been replicated many times (cf. Ansorge, Klotz,& Neumann, 1998; Klotz & Neumann, 1999; Klotz & Wolff, 1995), with different shapes, in different laboratories (cf. Eimer & Schlaghecken, 1998; Enns & Di Lollo, 1997; Jaśkowski, van der Lubbe, Schlotterbeck, & Verleger, 2002; Leuthold & Kopp, 1998; Vorberg, Mattler, Heinecke, Schmidt, & Schwarzbach, 2004; Woodman & Luck, 2003), and with a feature different from shape (e.g., color; Ansorge, Becker, & Breitmeyer, 2009; Ansorge, Breitmeyer, & Becker, 2007; Breitmeyer, Ogmen, & Chen, 2004; Breitmeyer, Ro, & Singhal, 2004; Schmidt, 2002; Vath & Schmidt, 2007). Unconscious visual feature selection of successfully masked stimuli provides a strong argument for the temporal precedence of feature selection over the completion of orienting.

## Is orienting conditional on visual feature selection?

Going one step further, the strongest arguments for a temporal pre-cedence of feature selection over orienting came from studies showing that orienting is even conditional on prior feature selection. A test of this sequence requires fulfillment of three conditions, as these were paradigmatically defined in so-called contingent capture experiments (Folk, Remington, & Johnston, 1992). *Contingent capture of attention* denotes a form of orienting that is conditional on a match between top-down controlled templates for relevant visual features and actually selected visual features of an input stimulus. According to this concept, only stimuli with a feature matching to the search template elicit orienting towards their position. It is clear that the very concept of contingent capture requires that features, such as color or shape of a stimulus, can be selected prior to orienting towards this stimulus.

The first of the three conditions that needs to be fulfilled to demonstrate contingent capture is that the researcher has to have a motivated hypothesis about the content of the search templates used by the participants. This is usually ensured by the task and the instructions. The researcher can ask participants to search for a class of targets defined by a particular feature. For instance, the researcher can inform the participants that the only red stimulus in the display is the target. In addition, the researcher can take more or less care that the participants can only find the target by the particular instructed feature. If less care is taken and the target can be found by more than one of its characteristics (e.g., if it can also be found as the only circular stimulus in the display), the researcher’s assumptions about the content of the search template could be wrong, and search templates could vary across participants or time. (An important side condition for contingent capture experiments is thus again the participants’ uncertainty about the target position.)

The second and the third condition to be met pertain to the use of two sorts of irrelevant cues (or distractors): cues with a template-matching feature (the “matching cues”) and cues without a template-matching feature (the “non-matching cues”). Importantly, these cues must be fully irrelevant for the task. In particular, the cues should not inform about the likely target position, because otherwise participants have good reasons to search intentionally for the cues in addition to their intentional search for the relevant targets. Intentional search for the cues is usually disencouraged by ensuring that the positions of the cues and the positions of the targets are uncorrelated across trials or by using a number of different possible target positions larger than two and by never presenting the cue at the target’s position. To demonstrate contingent capture, it then needs to be shown that participants do orient towards the matching cue but not towards the non-matching cue. This result would confirm that the search template for a target feature, say its color, is also a necessary precondition of the capture by the matching cue which happens to have the same searched-for feature, say a particular color.

 In their ground-breaking study, Folk et al. (1992) turned a crucial but untested premise of some stimulus-driven research approaches (cf. Mulckhuyse & Theeuwes, 2010b) into a hypothesis stating that orienting could be feature-specific rather than feature-unspecific (i.e., rather than be driven by abrupt onsets or by any singleton). Folk et al. (1992) tested whether participants could use visual features, such as stimulus color, to carefully select only stimuli that were probably targets. They used two kinds of defining target features that their participants had to search for in different blocks. In one block, the targets were defined by a particular color (i.e., red) and as a color singleton (i.e., the other elements of the target display were white). In an alternative block, the targets were defined by their status as an abrupt-onset singleton. The participants’ task was to search for the target by these pre-specified features and to report the target’s shape. 

 The reasonably well motivated hypothesis of Folk et al. (1992) concerning the content of the search templates was that participants either searched for a color target (in the blocks in which all targets were red) or for an abrupt-onset target (in the blocks in which all targets were abrupt-onset singletons). In each of the blocked conditions, Folk et al. used matching cues (a red cue presented shortly before a red target, or a white onset-singleton cue presented shortly before a white onset-singleton target) and non-matching cues (a red cue presented shortly before a white onset-singleton target, or a white onset-singleton cue presented shortly before a red target). Cues as well as targets were presented with equal probabilities at each of four locations, with cue and target locations uncorrelated across trials. 

 The predictions of the contingent capture view and, hence, the assumption that feature selection precedes orienting, were fully confirmed. A matching cue at the same position (SP) as the target facilitated the search for the target and the correct responses to it. This was in comparison to a matching cue at a different position (DP) than the target. A non-matching cue, however, led to about the same search time and response speed in SP and DP conditions. Folk et al. (1992) concluded that orienting was conditional (or contingent) on feature coding and a match between stimulus features and search templates, although some instances of bottom-up capture seem to require yet another explanation than contingent capture (cf. Burnham & Neely, 2008). 

 Conclusion: Neither abrupt-onset singletons, nor color singletons were special in their status as exogenously summoning attentional capture. Both singleton effects were over-ruled by feature-contingent orienting (see also Ansorge & Heumann, 2003, 2004; Ansorge, Kiss, Worschech, & Eimer, 2011; Folk & Remington, 1998; for a review, see Burnham, 2007). Subsequent research additionally showed that endogenous control principles can also account for singleton capture. Bacon and Egeth (1994) showed that participants endogenously search for singletons in general (i.e., stimuli that differ in any of their features from their surrounds) if such an endogenously controlled search template allows finding all targets, but that participants switch to an endogenous feature-search mode if the targets are non-singletons. This means that Folk et al. (1992) tested their feature-contingent orienting hypothesis under very conservative conditions, because endogenous search for singletons was also possible in Folk et al. (1992). This also means that one cannot simply infer a general precedence of exogenous orienting over feature selection from evidence for singleton capture (Leber & Egeth, 2006). Below it will be explained that this is crucial for an appropriate understanding of what happens during unconscious orienting. 

### Deallocation

 There is a caveat to the argument of Folk et al. (1992). It could be that failures to reveal orienting by non-matching cues are due to fast orienting, followed by fast reorienting. On this account, participants oriented towards both, the matching and the non-matching cue (cf. Mulckhuyse & Theeuwes, 2010b; Theeuwes, Atchley, & Kramer, 2000). Thereafter, however, participants could have used the small interval between cue and target for a quick deallocation from the non-matching cue, reasonably ahead of the presentation of the target. As a consequence, zero orienting towards the non-matching cue would have been falsely suggested by the lack of a cueing effect only because orienting had already reverted to a neutral position (equally distant from all potential target positions). In addition, in the matching condition, a high cue-target similarity might have required more time to discriminate the cue from the very similar target before the cue could be rejected as irrelevant. As a consequence, participants would not have deallocated their attention in the matching condition at the time when the target had its onset. Therefore, orienting (as a cueing effect) was found in the matching condition only. 

 Some authors claim to have found positive evidence for deallocation and/or exogenous orienting. Belopolsky, Schreij, and Theeuwes (2010) reported that their participants oriented towards uninformative non-matching color cues. True, this finding is at odds with that of Folk et al. (1992). The study of Belopolsky et al., however, does not necessarily demonstrate exogenous orienting. Participants could well have endogenously searched for all targets as singletons. We have explained this in more detail above. There is also no evidence that participants oriented towards the uninformative non-matching cue early after its onset. The effect of the non-matching cue could have also occurred at a later time after the cue’s onset. 

 Another study by Schreij, Owens, and Theeuwes (2008) is also equivocal in this respect: In that study, adding one placeholder element in the target displays in the Folk et al. (1992) paradigm increased search times. Schreij et al. regard this as evidence that the new element must have captured attention. Yet, it is a standard finding in the visual search literature that additional stimuli in the display increase search time (cf. Duncan & Humphreys, 1989; Treisman & Gelade, 1982; Wolfe, 1994). Maybe Schreij et al. think it is noteworthy that an additional placeholder increased search time although the participants oriented towards the matching cues. This line of thinking, however, presupposes that the matching cues attracted attention in a deterministic manner, in all trials, and to a maximal extent. If, however, orienting towards the matching cue shows variability as is typical for almost all mental processes, and if orienting effects were therefore less than maximal, there would have been ample air for an additional delay of the target search time imposed by one more placeholder in the target display. 

 Data by Pratt and McAuliffe (2002) are perfectly in line with this suspicion. These authors showed that orienting towards a matching cue is less than optimal at the time of the targets because with time (during the cue-target interval) participants withdraw attention even from the matching cues (cf. Posner & Cohen, 1984). Accordingly, an additional placeholder could well have delayed search times despite of overall net orienting to the matching cues in Schreij et al.’s study. 

#### Exhaustive measures of feature-dependent orienting

What is needed to test the possibility of deallocation is an exhaustive measure of orienting. One has to continuously track orienting during a trial and right from stimulus onset onwards to find out whether feature-independent (singleton-driven) orienting precedes feature-dependent orienting. Continuous tracking of orienting towards a stimulus, from its onset and with millisecond resolution is possible with event-related potentials (ERPs). ERPs should therefore be sensitive to initial exogenous orienting, if it exists. When ERP measures do not respond to non-matching singleton cues but only to matching singleton cues, the hypothesis that exogenous orienting (mandatorily) precedes feature coding and is just camouflaged by deallocation is weakened.

Noteworthy, the large majority of ERP studies failed to find any evidence whatsoever for early exogenous orienting with non-matching cues (cf. Eimer & Kiss, 2008; Eimer, Kiss, Press, & Sauter, 2009; Kiss, Jolicoeur, Dell’Acqua, & Eimer, 2008). Instead in one study, feature-dependent orienting in ERPs was found even within 100 ms after cue onsets (Zhang & Luck, 2009).

 In addition, Ansorge et al. (2011) used ERPs as an exhaustive measure to test whether exogenous orienting towards non-matching cues can be as strong as feature-specific top-down contingent orienting if there is no incentive to deallocate attention away from the cues. For that purpose, Ansorge et al. (2011) presented top-down matching singleton color cues as well as non-matching singleton color cues at the target’s position in 100% of the trials. Under these 100% SP conditions, there was absolutely no incentive for the participants to withdraw attention from any of the cues. If it would be true that faster deallocation after non-matching than matching cues falsely suggested more initial top-down contingent feature-specific orienting in prior studies, one would have expected no difference between the ERP orienting effects under the conditions with 100% SP cues. However as in other studies, top-down contingent feature-specific attentional capture was stronger than exogenous singleton capture. This result makes clear that stronger deallocation is not a plausible explanation for the results of the study of Folk et al. (1992). 

 Two other ERP studies appear to be in line with the deallocation account at a first glance. Hickey, McDonald, and Theeuwes (2006) found early orienting to a non-matching cue. In that study, however, the task does not disencourage an endogenous search for singletons. Ansorge and Heumann (2006) found early lateral ERP effects of non-matching unconscious cues. However, in their study, ERP effects are best explained by sensory differences rather than attentional effects because there was no behavioral orienting effect (Ansorge & Heumann, 2006).

## Unconscious orienting

How does orienting operate in the case of unconscious visual stimuli? Basically, the two options discussed above have also been studied with unconscious visual stimuli: exogenous orienting preceding feature selection on the one hand, and endogenous feature-contingent orienting on the other.

### Endogenous feature-contingent orienting towards unconscious stimuli

 Several studies demonstrated endogenous feature-contingent orienting towards unconscious visual stimuli. Ansorge and Neumann (2005) used black or red targets and found that their participants only oriented towards unconscious black primes, if they searched for black targets but not if they searched for red targets. Scharlau and Ansorge (2003) used matching singletons and non-matching singletons as masked primes, and demonstrated that perceptual latency priming was stronger with matching than with non-matching primes, although participants could have also searched for all targets by endogenous singleton search alone. Held, Ansorge, and Müller (2010) equally found that if participants searched for visible color singletons, participants oriented towards a masked color singleton prime whereas they did not orient towards a masked shape singleton prime. 

Again, we have to ask whether deallocation can be ruled out with a more exhaustive ERP measure. This was done by Woodman and Luck (2003; for related results, see also Jakowski et al., 2002). These authors found that if a masked matching stimulus and a masked non-matching stimulus were presented concomitantly, one left and the other right of fixation, participants oriented towards the matching stimulus. A possible weakness of Woodman and Luck’s (2003) study is that it showed a net advantage in orienting for the matching cue over the non-matching cue. Because the matching and the non-matching cues were pitted against one another, it is possible to overlook orienting towards the non-matching singleton in the net orienting effect created by both stimuli. Later studies to be discussed below (Ansorge, Horstmann, & Worschech, 2010), however, are not open to this criticism.

 An alternative procedure to test orienting towards masked unconscious color singletons was developed by Ansorge, Kiss, and Eimer (2009). These authors presented one masked matching non-singleton color prime per trial and found evidence for orienting. The effect was also found in the ERPs during so-called nogo trials. In the nogo trials, only a cue was shown and no color target was presented. The nogo trials therefore clearly demonstrated that the feature-specific top-down contingent orienting effect was produced by masked stimuli alone. Importantly, evidence for orienting was found regardless of whether or not a target with the same color as the matching prime was presented to the participants in the trial before. This finding makes clear that endogenous feature-dependent orienting explains the effect exclusively, with no contribution of bottom-up priming of pop-out as an alternative exogenous origin of the effect (cf. Belopolsky et al., 2010; Maljkovic & Nakayama, 1994). 

 Following up on orienting effects in ERPs of masked unconscious visual color cues (cf. Ansorge, Kiss, & Eimer, 2009), Ansorge et al. (2010) presented only one singleton cue per trial. In half of these trials, this was a matching cue and in half of the trials it was a non-matching cue. Under these conditions, orienting towards the unconscious non-matching singleton would not be camouflaged by simultaneous orienting towards a matching singleton, as was discussed above with reference to Woodman and Luck (2003). Nonetheless, the conclusions of Woodman and Luck were fully supported. The only evidence for orienting was found in ERPs towards masked matching color singletons. No such orienting was found towards non-matching color singletons. In addition, Ansorge et al. (2010) tested whether the orienting effect of masked matching cues is at least stronger if the cue is a singleton than if it is a non-singleton (cf. Lamy & Zoaris, 2009). However, no difference was found between the behavioral cueing effects in these two conditions – that is, the singleton status of the masked cues even failed to boost orienting towards these stimuli if they matched the top-down search sets by their particular feature. In other words, a feature-match was not only necessary for an orienting towards the masked color cues, it was also the only statistically reliable origin of orienting that could be found. 

 The top-down contingency principle in attentional control also generalizes to overlearned symbols. Reus, Pohl, Kiesel, and Kunde (2011) used visible as well as masked arrows as cues. These authors found that visible arrows elicited attention shifts in target-predictive and non-predictive conditions. By contrast, masked cues only elicited attention shifts if the cues were also predictive of the likely target positions. According to the authors, the participants had to set up an intention to process the visible arrows for an attentional effect of the masked arrow cues. Only when an informative visible arrow cue was used but not when a non-predictive arrow cue was used, participants set up this intention. As a consequence of this intention, masked arrow cues that were presented in a sequence of trials randomly inter-mixed with the informative visible arrow cue led to an attention shift. 

### Exogenous orienting towards unconscious singletons

Other authors come to an opposite conclusion and claim to have demonstrated exogenous orienting towards unconscious singletons (e.g., Ivanoff & Klein, 2003; McCormick, 1997; Mulckhuyse & Theeuwes, 2010a). Among the most convincing demonstrations of exogenous orienting is the research by Mulckhuyse and her colleagues (e.g., Mulckhuyse et al., 2007). Yet even this research fails to convincingly demonstrate exogenous orienting, as will be detailed below.

 The basic paradigm has been developed in Mulckhuyse et al. (2007). These authors used placeholders at three different positions, and one of these placeholders started a little earlier than the others. This was the onset-singleton cue. Participants had difficulties discriminating which of the three placeholders was presented first and, therefore, the temporal onset-singleton cue was considered unconscious. After the cue and placeholders, an onset-singleton target appeared within one of the placeholders with the position of cue and target being uncorrelated over trials. With this setup, Mulckhuyse et al. (2007) found a cueing effect better performance for SP conditions than DP conditions with a brief cue-target interval. 

 From the above, however, it is clear that this study fulfills all criteria necessary for contingent capture. The targets were onset singletons, and the masked cues were onset singletons. Thus, a search set for the relevant features can account for the orienting effects found by Mulckhuyse et al. (2007). No need to revert to an exogenous orienting effect. 

### Speed of orienting in unconscious cueing

Another criterion of exogenous unconscious orienting that has been advocated in the past is the speed of the orienting effect (Mulckhuyse & Theeuwes, 2010b). Above we have reviewed that quick orienting was sometimes considered to be typical of exogenous orienting (cf. Müller & Rabbitt, 1989; Ogawa & Komatsu, 2004). This speed criterion was used to argue that orienting towards unconscious onset cues during early phases of the saccadic trajectory must have been of an exogenous origin (cf. Van der Stigchel, Mulckhuyse, & Theeuwes, 2009).

Yet speed is an equivocal criterion of exogenous orienting because contingent capture as one form of endogenous feature-dependent orienting is also fast (Bichot, Rossi, & Desimone, 2005; Zhang & Luck, 2009). Endogenous orienting in the form of contingent capture effects can be found with simultaneous onsets of matching cues and targets, and the orienting effect is present among the quickest responses, when no exogenous orienting effect is observed (cf. Ansorge & Horstmann, 2007; Ansorge, Horstmann, & Carbone, 2005). This means that exogenous unconscious orienting cannot be concluded by reverting to a speed criterion. The same conclusion is supported by the earlier onset of orienting effects in ERPs of top-down matching color cues than of non-matching singleton cues (cf. Ansorge et al., 2011).

## Summary and discussion

In the present review, we have carefully summarized the general conditions that must be fulfilled by an experimental protocol for demonstrating endogenous orienting to visual stimuli, for exogenous orienting, and for orienting to unconscious visual stimuli. We have shown that there is only one fail-safe criterion by which it can be concluded with certainty that an orienting effect reflects exogenous orienting: the absence of a fitting endogenous feature-specific search criterion as a precondition for an orienting effect (cf. Folk et al., 1992). We think that some studies that used conscious cues for orienting successfully pass this criterion and thus support exogenous orienting (e.g., Burnham & Neely, 2008).

More importantly, however, our review of the major evidence in the domain of unconscious orienting has shown that endogenous orienting is the rule in these studies and that exogenous orienting has not yet been demonstrated with unconscious stimuli. None of the studies that we reviewed fulfills the requirement of demonstrating unconscious orienting in the absence of a fitting search template. In the few studies where the relevance of a match between the unconscious stimulus and the search template was manipulated, the match between unconsciously presented visual features and search set was found to be necessary for orienting (e.g., Woodman & Luck, 2003).

 A few limitations of our review are noteworthy. We have not discussed work with patients. To our knowledge, however, the conclusion would be the same. Kristjánsson, Vuilleumier, Malhotra, Husain, and Driver (2005), for example, demonstrated in two patients with visual neglect of the right visual hemifield that an undetected singleton color presented to the neglected side in trial n exogenously primed orienting towards a similar color stimulus in a subsequent trial n + 1. Note that this means that the unconscious singleton in trial n was chosen in accord with the patients’ top-down search template for a singleton. Likewise, patients with a scotoma in V1 who fail to report a visual stimulus in their blind field can orient towards the invisible stimuli, a condition termed *blindsight* (e.g., Weiskrantz, 1986; Weiskrantz, Warrington, Sanders, & Marshall, 1974). Yet, these patients endogenously search for these stimuli in their blind field. Thus, again, we have good reason to consider these effects as a form of endogenous rather than exogenous orienting. 

We have also not discussed the underlying physiological substrate of unconscious visual effects in the brain. We believe that a system encompassing the superior colliculus, posterior parietal cortex (PPC), and frontal eye fields could be part of that substrate (cf. Ansorge, 2003; Mulckhuyse & Theeuwes, 2010a; Weiskrantz, 1986). Where to finally put the origin of unconscious orienting in this network of interconnected areas remains yet to be seen. However, we believe that PPC is a likely candidate for a role of unconscious vision in orienting because of PPC’s involvement during unconscious visually guided action control in general (cf. Goodale & Milner, 1992) and in the programming of saccades (e.g., Bueno & Andersen, 2006) and endogenous contingent orienting in particular (e.g., Ogawa & Komatsu, 2009).

Finally, the way that the studies reviewed in the present article measured visibility varied, and we have not weighed the reviewed evidence from the studies by the sophistication of the methods used to secure that the stimuli or features were truly unconscious (cf. Eriksen, 1960; Holender, 1986; Reingold & Merikle, 1988). We used a relatively liberal criterion for the inclusion of studies. This was done in the interest of a maximally encompassing review. Conclusions, however, would be no different with a more stringent criterion for unconscious presentation.

### Conclusion

We have started with examples of very primitive organisms, such as nematodes that orient towards the source of light, probably without any awareness of the light or consciousness about it. We think that these primitive organisms provide good examples concerning the origin and ultimate function of unconscious orienting. During evolution, animal species predating humans had to develop more flexible sensorimotor mechanisms in the service of coordinating their actions within a dynamically changing environment (cf. Allport, 1987; Brembs, 2011; Neumann, 1987). Orienting is just one of these sensorimotor mechanisms.

What is important in this context is that in order to be adaptive, these orienting mechanisms should be endogenously and flexibly steerable and not only be exogenously driven by some enduring visual properties of the environment. To be adaptive an orienting mechanism has to be steerable by different visual features, depending on the kind of intended action to be executed and on the particular action goal pursued. Under this perspective, orienting, as we see it, has become ever more endogenously controlled in animals and has been also a precursor and building block of conscious vision both in evolutionary terms and in real-time (cf. Lamme, 2003). Endogenous orienting serves the purpose of highlighting relevant features and of down-weighting irrelevant features, either for action control or, in some species, for a conscious visual representation of the environment.

## Footnote

1 Attention and awareness can be dissociated: An observer’s attention can be directed to a stimulus, without a subsequent aware perception of this stimulus (cf. Lamme, 2003). Much of the evidence in this article concerns this fact (e.g., Ansorge, Kiss, & Eimer, 2009). However, the opposite pattern of dissociation is difficult to demonstrate and accordingly has not been demonstrated so far: awareness in the absence of attention.
